# Better late than never: defining the ideal vaccine that targets pre-erythrocytic malaria infection

**DOI:** 10.1038/s44321-025-00300-9

**Published:** 2025-09-29

**Authors:** Grace M Rochfort Peters, Jake Baum

**Affiliations:** School of Biomedical Sciences, UNSW Sydney, Kensington, NSW 2052 Australia

**Keywords:** Immunology, Microbiology, Virology & Host Pathogen Interaction

## Abstract

J. Baum and G.R. Peters discuss vaccine efficacy and mechanism of protection of PfSPZ co-administered with a single therapeutic dose of the chemoprophylactic drug combination atovaquone-proguanil as reported by S. Borrmann and colleagues, in this issue of *EMBO Mol Med*.

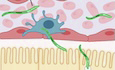

## Teaser

Progress towards a much-needed malaria vaccine has accelerated in recent years. The most significant recent event was the independent licensure of the first two subunit vaccines against malaria, RTS,S/AS01 (Mosquirix^TM^) from GSK and R21/Matrix-M from the Serum Institute of India, which received full World Health Organisation (WHO) prequalification in 2022 and 2023, respectively. Both vaccines require a primary series of four doses to confer maximum protection to children against the most virulent species of malaria caused by *Plasmodium falciparum*. This immunisation series, given over a 12 to 18-month period, elicits an immune response that targets the free parasite form—called a sporozoite—during its journey from mosquito bite, through the dermis and on to the liver, where it infects hepatocytes. It elicits a predominantly B-cell response (with a modest CD4^+^ T-cell response) specifically aimed at the circumsporozoite protein (CSP), the main sporozoite surface protein, aiming to block parasite colonisation of the liver. Whilst the licensure of these two vaccines heralds a landmark in global health, the level and durability of protection each confers is still uncertain, especially in infants and in areas of perennial, high-intensity transmission. Rather than quell enthusiasm for further vaccine development, however, this benchmark has bolstered efforts to find alternative strategies that complement or improve on overall vaccine efficacy and longevity. This enthusiasm is still urgent, with over half a million children under 5 still dying each year from malaria (Venkatesan P, 2025).

**Figure 1:**
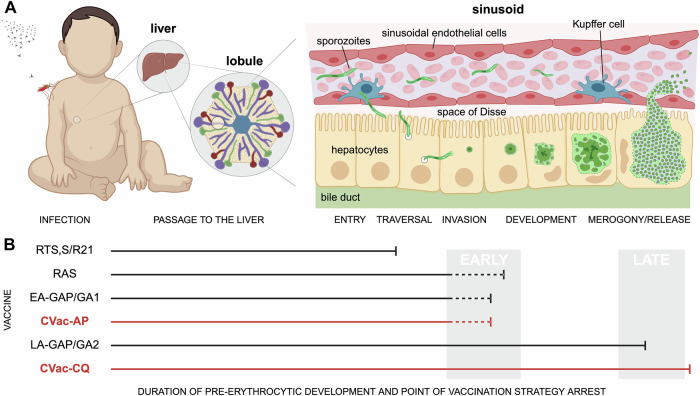
Vaccine targeting across the malaria parasite liver stages. (**A**) An overview of the journey of the infectious sporozoite from mosquito bite through the dermis, circulatory systems to the liver sinusoid where the parasite at first traverses and then eventually invades a resident hepatocyte. (**B**) A schematic of the duration of pre-erythrocytic development through which parasites progress (marked by a flat-ended arrow) under different vaccination strategies: RTS,S/R21, radiation attenuation (RAS); chemoprophylaxis cover (Chloroquine, CQ or Atovaquone/Proguanil, AP); or genetic attenuation (Early-Arresting Genetically Attenuated Parasites, EA-GAP/GA1 versus Late-Arresting LA-GAP/GA2). Note there is still uncertainty around the precise timing of arrest in the early-arresting forms (e.g., Suhrbier et al, 1990), hence dotted lines. The current study by Borrmann et al reinforces the value of using a vaccine strategy that permits a longer duration of intra-hepatic development to gain maximal immune protection, though the precise mechanisms that define improved protection, and the optimal window are still uncertain. Created in BioRender. Baum J (2025) https://BioRender.com/obe6oed.

The best efficacy to date for any malaria vaccine has come from immunisation with a live parasite. The appeal of live sporozoite vaccines in particular—abbreviated to PfSPZ for *P. falciparum*, the most virulent human-infective species—is clear. Given the sporozoite’s role in establishing asymptomatic liver infection (Fig. 1), vaccines that target pre-erythrocytic development can deliver a robust humoral and cellular immune response, and, if attenuated, will do so without exposing the host to the clinically significant blood stages of disease that follow. Much of the key progress made with an attenuated PfSPZ vaccine approach has come from trials with radiation-attenuated sporozoites (RAS), first demonstrated to be protective against homologous challenge five decades ago (reviewed in (Richie et al, 2023)), which has since seen substantial clinical progress in adults (Diawara et al, 2024; Sirima et al, 2022), though less so in infants (Oneko et al, 2021). Beyond RAS, work undertaken by Roestenberg and colleagues demonstrated that sporozoite immunisation delivered under the cover of treatment with the drug Chloroquine (CQ) could confer a remarkable level of sterile protection (Roestenberg et al, 2009). Referred to as chemoprophylaxis vaccination (CVac for short here), coverage of PfSPZ delivery with CQ, a drug that targets the blood stages specifically, allows the liver stage of the parasite to develop freely, but mops up any parasites that break through to the blood stage. Not surprisingly, this approach has been evaluated further in malaria-endemic populations. However, field trials have been variable and generally less successful than results observed in malaria-naïve individuals (Coulibaly et al, 2022). Reasons for the suboptimal response observed are unclear, but given the substantial compliance required for administering CQ before and after immunisation, plus concerns about drug tolerability, many researchers have asked what alternatives might improve the CVac approach (Richie et al, 2023).

In this issue of *EMBO Molecular Medicine*, Borrmann et al have addressed this question, exploring whether the practical and safety advantages of the well-tolerated drug combination atovaquone/proguanil (AP) might provide the answer. AP, a fixed-dose antimalarial marketed as Malarone^TM^, acts on both liver and blood stages by disrupting mitochondrial function (atovaquone) and inhibiting DNA synthesis (proguanil), making it a promising alternative for safer sporozoite attenuation. Previous studies have already established AP’s ability to arrest liver-stage development in the rodent malaria model, *P. berghei*, and prevent blood-stage infection in humans when given prophylactically. Given these properties, AP could be administered at the time of immunisation, eliminating the risk of breakthrough malaria but preserving liver-stage antigenicity to elicit a fully protective immune response.

Bormann and colleagues lead off their study with in vitro work, demonstrating clearly that a single dose of AP or atovaquone alone can induce a robust early arrest of liver-stage parasites in hepatoma cultures without impacting sporozoite entry. These arrested parasites persisted intracellularly for days, with a phenotype similar to other attenuation methods like RAS (Suhrbier et al, 1990)—though the differences in their exact timing of arrest is unclear. When evaluated in live rodent studies, 97% of C57BL/6 mice showed no breakthrough blood stage infection after receiving 10,000 intravenous sporozoites co-administered with either atovaquone or AP, again supporting previous observations that this drug would provide chemically induced cellular arrest in the liver. Following this, 88–100% (sterile) protection was then observed against subsequent mosquito challenge after a two- or three-boost immunisation regime, respectively. Given that pre-exposure of sporozoites to AP confirmed the drug’s action targets liver stages, not the sporozoites themselves, this provided a powerful preclinical rationale for moving the regimen to human trials, especially given the robust CD8⁺ T-cell responses and high anti-sporozoite antibody titres against CSP seen.

However, in contrast to the rodent work, a phase I clinical trial in humans found that while AP drug coverage completely blocked liver-to-blood stage progression—preventing parasitaemia in all PfSPZ vaccine recipients—the resulting protection against controlled human malaria infection was minimal (20–25%), markedly lower than a similar regimen previously reported with CQ cover. The detail here is important and highlights why moving these observations from rodent studies to human clinical trials is critical, even if the outcome was disappointing (in terms of AP’s usage for CVac). In the trial, healthy malaria-naïve volunteers aged 18–45 were immunised with either 50,000 or 150,000 sporozoites *via* direct intravenous injection, three times at four-week intervals, alongside a single oral dose of AP (1000 mg/400 mg) given within one hour. This dose is four times higher than the chemoprophylactic dose but equivalent to a standard three-day treatment course. The lower sporozoite number group matched the same group’s previous CVac-CQ study (Mordmuller et al, 2017). Notably, antibody titres against the CSP protein were found to be lower in the 50,000 sporozoite group receiving AP than they were in the previously reported 50,000 group receiving CQ. Whilst the group receiving AP with 150,000 sporozoites did have much higher CSP titres, as described, the key finding was that this did not enhance efficacy.

Taken together, the modest efficacy of CVac-AP compared to CVac-CQ points a very strong finger of blame at the importance of CSP-independent mechanisms—especially cellular immunity—in mediating protection. Work in both rodent and human studies supports this (Butler et al, 2011; Lamers et al, 2024). For example, human clinical trials with early-arresting genetically attenuated *P. falciparum* sporozoites (EA-GAP or GA1 parasites) compared with late arresting (LA-GAP/GA2) confirmed you get much better protection the later the liver-stage parasite arrests (Fig. 1). What’s important to note is that whilst similar levels of CSP antibody levels were seen in EA-GAP/GA1 and LA-GAP/GA2 arms of the *P. falciparum* trial, the efficacies of the two strategies couldn’t be more striking: 12% versus 89% (Lamers et al, 2024). Thus, whilst sporozoite-dependent protection may not be purely driven by a cellular (likely CD8 + T cell) response, it certainly plays a critical part.

The final insight from the study by Borrmann et al relates to the thorny question of the non-CSP response. Antibody profiling from serum revealed that sporozoite-immunised individuals under AP coverage demonstrated a marked reduction in antibody production against two key liver-stage antigens, LISP2 and LSA1, compared to those who received CQ. Similarly, CQ recipients showed higher anti-MSP5 titres than AP equivalents. Thus, whilst CSP titres look less and less reliable as direct correlates of PfSPZ immunisation protection, these other antigen-specific antibody responses may still serve as key biomarkers for future studies looking for a still elusive correlate of protection.

The observations from the current study and growing literature around chemo- and genetically attenuated parasite vaccination suggest a clear line of attack, though questions remain. If we want to harness the power of whole sporozoite vaccination, particularly using CVac, we should choose strategies that arrest liver-stage development as late as possible (Fig. 1). Liver schizonts that never commence replicative stages (as in RAS, EA-GAP/GA1, and CVac-AP) appear unable to confer full protection. This may be a matter of timing, i.e., long enough to facilitate a more potent CD8 + T-cell response (Butler et al, 2011) or the superior immunogenicity and/or breadth of antigens present in the late liver stage. The duration of the arrested liver stage may also explain some of the discrepancies that confound translation of rodent to human studies. Whilst the kinetics of T-cell maturation/activation may be similar between the two species, the timing of parasite development are markedly different (2–3 days in rodent malaria versus 6–7 in humans). T cells clearly have less time in mice. In short, it seems for pre-erythrocytic vaccines it is better to be late (in development) rather than early or never at all. Perhaps even better still, strategies that can identify the antigens responsible for the success of late-arresting protection, both non-CSP and cellular, may yet deliver a subunit or even mRNA vaccine that has all the bang of PfSPZ, but without the inherent complications of delivering a live infectious parasite (Borrmann et al, 2025).
